# Exposure to Air Pollution and Changes in Resting Blood Pressure from Morning to Evening: The MobiliSense Study

**DOI:** 10.3390/ijerph22060872

**Published:** 2025-05-31

**Authors:** Lisa Sekarimunda, Clelie Dureau, Basile Chaix, Sanjeev Bista

**Affiliations:** 1 Sorbonne Université, INSERM, Institut Pierre Louis d’Epidémiologie et de Santé Publique IPLESP, Nemesis Team, Faculté de Médecine Saint-Antoine, 27 rue Chaligny, 75012 Paris, Franceclelie.dureau@iplesp.upmc.fr (C.D.); basile.chaix@iplesp.upmc.fr (B.C.); 2 Centre de Recherche en Santé Publique, Université de Montréal, 7101 Avenue du Parc, Montreal, QC H3N 1X9, Canada

**Keywords:** sensors, air pollution, noise, blood pressure, multilevel models, mixture models

## Abstract

Several epidemiological studies have documented associations between air pollution exposure and cardiovascular responses, including adverse effects of air pollutants on blood pressure (BP). However, previous studies only considered the effect of specific air pollutants on resting BP, and did not sufficiently consider the independent effects of various air pollution species as well as their overall mixture effect. We addressed this gap in our MobiliSense sensor-based study among 273 participants living in the Grand Paris region. Participants wore personal monitors to assess personal exposure to particles [black carbon and particulate matter smaller than 2.5 μm in diameter (PM_2.5_)] and gaseous pollutants [ozone (O_3_), nitrogen monoxide (NO), carbon monoxide (CO), and nitrogen dioxide (NO_2_)] along with noise exposure. Participants were asked to measure their blood pressure (BP) at rest in the mornings and evenings for three days. Multilevel models with a random intercept at the individual level explored the relationship between air pollution exposure (averaged over the day) and change in resting BP from morning to evening. We also used the quantile G-computation method to estimate the joint effect of the mixture of targeted air pollutants on resting BP. Sensitivity analyses examined the associations between air pollution exposure averaged at different temporal scales before evening BP measurements and the outcome. A quantile increase in the mixture of air pollutants (PM_2.5_, NO_2_, NO, CO, and O_3_) over the day did not affect changes in systolic BP [−0.33 mmHg (95% CI: −3.31, 2.65)] and diastolic BP [−0.53 mmHg (95% CI: −2.66, 1.60)] from morning to evening. When shorter time exposure windows were considered (from a few minutes to a few hours), both NO and the mixture showed positive associations with the morning-to-evening DBP change in only some of the models. Future studies with sufficient repeated BP measurements for more participants should test the association at varying temporal scales (minutes to days) to better understand how air pollution exposure influences resting BP.

## 1. Introduction

High blood pressure (BP) has been identified as one of the major contributors to cardiovascular morbidity and mortality [1,2]. It is believed that a large number of repeated episodes of acute increase in BP may lead to hypertension [3], primarily among the susceptible populations. It is reported that a 20 mmHg increase in systolic BP (SBP) or even a 10 mmHg increase in diastolic BP (DBP) elevates the cardiovascular mortality risk by more than two-fold [4,5].

In empirical studies, exposure to air pollution, over the short and long term, has been found to be associated with the initiation of cardiovascular events through changes in blood pressure [6]. Even modest increases in airborne pollution could trigger an increase in arterial BP within hours [6]. Behavioral factors added to genetic background lead to hypertension, partially through changes in BP initiated by exposure to air pollution [7]. Experimental studies have also confirmed various biological pathways through which air pollution exposure elevates BP, including oxidative stress, vasomotor dysfunction, altered autonomic function of the heart, induction of systemic inflammation, and endothelial dysfunction [8,9]. While some empirical studies have assessed relationships between air pollution and ambulatory BP in order to understand cardiovascular conditions [10,11,12,13], it is considered that lowering the average resting BP can have a substantial impact on decreasing mortality from coronary heart disease and stroke by 4% to 6% [14].

Several past studies have documented a positive association between day-level air pollution exposure and resting blood pressure [15,16,17,18,19]. However, not all the studies by far have used personally measured air pollution exposure to estimate its effect on BP, leading the associations to be biased due to exposure misclassification occurring from using station-monitored data as a proxy of the real exposure [20]. In addition, none of the past studies has assessed the change in morning to evening BP and how it is affected by the air pollution exposure in between the two or at different, shorter temporal scales before the evening measurement is recorded. Considering how exposure to air pollutants between a morning and an evening BP measurement explain the change over the day in BP is an appealing design to investigate the causal effect of air pollution on BP at rest. Exploring the change in BP over a day is relevant as a previous study has shown that the difference between morning and evening BP measurements is a crucial and independent predictor of stroke [21] and could affect clinical cerebrovascular disease [21]. Another methodological aspect still missing in this field of research is testing the joint effect of different air pollutants combined in a mixture on resting BP. Since people are exposed daily to mixtures of air pollutants depending on the different pollution sources in their environment, it is epidemiologically essential to estimate the joint effects of air pollutants with a mixture modeling technique, such as the quantile G-computation [22].

Addressing gaps in previous studies, the main aim of this study is to investigate the associations of air pollutant (PM_2.5_, CO, O_3_, NO and NO_2_) exposures, assessed at the personal level, over the day with changes in resting blood pressure from morning to evening using both a classical statistical analysis as well as a mixture modeling method. Furthermore, we evaluated the associations between shorter-term exposures to targeted air pollutants, averaged at minutes to hourly levels before the evening BP measurement, and changes in the morning to evening resting BP.

## 2. Materials and Methods

### 2.1. Study Population

Data for this study came from the MobiliSense study, which was conducted in the Grand Paris (the Paris City and some surrounding municipalities) in France, between May 2018 and October 2020 [13,23]. Eligible participants were recruited following a two-stage stratified random sampling procedure: the first stage involved the random selection of neighborhoods in the first and last quartiles of road traffic density in each quartile of area income [13,23]. The second stage involved randomly selecting dwelling units in the pre-selected neighborhoods from the 2013 and 2014 population censuses database (National Institute of Statistics and Economic Studies). Overall, 31,970 dwellings were selected from 234 neighborhoods. Postal mail was sent twice to invite residents from the pre-selected dwellings to participate in our study. People who planned to smoke during the study period or people who had at least one symptom or diagnosis of cardiovascular disease (heart failure, chest pain, etc.) or contagious lung diseases were not included in the study. Overall, 289 individuals participated in the sensor-based MobiliSense study [13,23,24].

### 2.2. Resting BP Assessment

Participants were asked to measure their BP at rest in the mornings and the evenings of days 2, 5, and 6 of our study. Regarding the morning measurement, the first decile, median, and ninth decile of the measurement time were 06:33 am, 7:42 am, and 09:33 am. The corresponding figures for the evening measurement time were 7:05 pm, 9:41 pm, and 11:27 pm. Each time, three successive measurements were taken (with 1 to 5-min intervals) while the participants were relaxed in a sitting position, with their non-dominant arm resting on a table. The average of those three successive BP measurements was considered to be the final BP measurement for each episode of measurement (morning or evening). The measurements were taken following the self-measurement protocol of the European Society of Hypertension [25]. Self-measurement of BP for several days is thought to be as reliable as BP measured at the physician’s office [24,26]. Participants used a certified blood pressure monitor (Nokia Health/Withings, Issy-les-Moulineaux, France) that permits a real-time transmission of BP measurements to a distant server through a connection to the smartphone provided to participants [24]. Participants were asked to answer a phone-based survey about the circumstances of BP measurement (medications taken, time spent at rest, social interactions, ambient noise) after each episode of measurement [24].

Our day-level outcome was defined as the change in the participants’ resting BP between the morning and the evening BP measurements of the same day for systolic and diastolic BP (SBP and DBP), as defined below.

Change in SBP (mmHg) = evening SBP (mmHg) − morning SBP (mmHg)

Change in DBP (mmHg) = evening DBP (mmHg) − morning DBP (mmHg)

### 2.3. Exposure to PM_2.5_, NO, NO_2_, CO and O_3_

Participants carried a Personal Air Quality Monitor (PAM) (Atmospheric Sensors, Bedfordshire, UK) on days 2, 5, and 6 to measure the breathing zone concentrations of gaseous pollutants (O_3_, NO_2_, NO, and CO) at a 10 s resolution expressed as part per billion (ppb). Likewise, PM_2.5_ concentration was measured at a 1 min resolution in µg/m^3^ [13,23] with the same device. The performance of the PAMs was characterized through outdoor co-locations at the end of the study, as described in Supplementary Section S1, following the methodology described in Chatzidiakou et al. 2019 [27]. These co-locations also permitted us to derive calibration equations for air pollutant concentrations, as explained in Supplementary Section S1. The device also measured ambient temperature and relative humidity. Personal exposures to all the air pollutants were averaged at various temporal scales, such as the exact window between the morning and evening BP measurements and from 5 min to 10 h before each evening BP measurements.

### 2.4. Accelerometry

Participants also carried an accelerometer (tri-axial wGT3X+, ActiGraph, Pensacola, FL, USA) on an inelastic waist belt over the 6 follow-up days. ActiGraph is one of the validated commercial accelerometers for accessing levels of physical activity [28]. We processed the accelerometer data by applying the low-frequency extension option instead of the normal filter in Actilife 6.13.3 to make it more adapted to slow-moving people [29]. A value of 0 for the 3-axes count for at least 1 h with a spike tolerance of 2 min of non-zero epochs, a default setting in Actilife, was selected to identify the non-wear time of the device [30]. The vector magnitude, as an indicator of physical activity, was calculated at a 10 s resolution as follows (the square root of the sum of squared activity counts) counts per minute:

$$VM=\sqrt{{\left(Axis1\right)}^{2}+({Axis2)}^{2}+{\left(Axis3\right)}^{2}}$$

Each 10-s epoch was classified as corresponding to moderate to vigorous physical activity by comparing its vector magnitude value to the threshold of 2690/6. We then calculated the percentage of time spent in moderate to vigorous physical activity (MVPA) [31,32] over similar time windows in which we aggregated the air pollution exposure. We took the percentage of MVPA over the period because the absolute length of MVPA would not be comparable between days since the time between the morning and evening BP measures differed from one day to another and may vary from person to person.

### 2.5. Activity Profiling

GPS data (at a 5 s resolution) were collected with BT-Q1000XT GPS receivers (QStarz, Tapei, Taïwan worn by the participants on the same belt as the accelerometer device throughout the study [24]. After the follow-up, GPS data were uploaded to the TripBuilder application, where they were analyzed with automated algorithms [24]. This application identified places visited by individuals, the trips they took, and the modes of transportation used during the trips [24]. The start and end times of each trip stage as well as the visited place obtained from the TripBuilder were further confirmed by research assistants with the participants during a phone mobility survey [24,33]. This mobility survey allowed us to add or correct the information on those trip segments or visited places that were missed or incorrectly captured by the GPS receiver. Using the SAS software (version 9.4), a detailed timetable was generated over the study period, incorporating places visited and trips subdivided into trip stages with corresponding times [24,33].

The proportion of time spent in the home, out-of-home, and in motorized and non-motorized transport (walking, biking, skateboarding, etc.) were calculated over the same time windows as for the air pollution exposure, using the timestamped information on visited places and trip segments (with information on modes) from the detailed timetable.

### 2.6. Noise Exposure

On days 3, 4, 5, and 6, the SV 104A dosimeter (Svantek, Warsaw, Poland) attached to the participants’ belt, with a microphone fixed at the collar near their ear, was used for monitoring personal noise exposure every second. The measured noise level was A-weighted [dB(A)] to capture sounds that correspond to human hearing, with frequencies ranging from 500 Hz to 8 kHz. Noise data were aggregated using the notion of equivalent sound level (Leq) in the corresponding time windows between morning and evening BP measures as well as in the windows before the evening measurement. Leq is defined as the constant sound that would have been generated with the same level of energy compared to the noise that was perceived during the time frame, expressed in dB (decibels) and computed as follows [13,23]:

$$L_{eq}=10\log\times \frac{1}{T}\int_{0}^{T}10^{\frac{L(t)}{10}}\;dt$$

*L_eq_*: equivalent sound level

*L*(*t*): noise level at time t

*T*: period length in seconds

#### Noise Prediction for Day 2

Personal noise exposure was not measured on the 2nd day of our study. Therefore, observed data from days 3 and 4 were used to predict the noise exposure for day 2 at the minute level. The method of predicting noise has been explained in our previous papers [13,23]. In brief, black carbon concentrations, mode of transportation and route of travel (when the person is travelling), activity place and activity type (when the person is in a certain place), week vs. weekend, time of the day (morning, noon, evening, and night) classified at the minute level for each participant were used in predicting noise exposure on days 3 and 4; the resulting equation was then applied to predict noise in day 2. Our study measured black carbon with Aethalometers (MicroAeth AE51, AethLabs, San Francisco, CA, USA) at a 10 s resolution and processed it with the optimized noise reduction averaging algorithm before aggregating data at the minute level. The detailed algorithm we applied in our study is cited in our previous publication [13,23].

In the end, noise was aggregated over the same time windows as for the air pollution exposure.

The R^2^ of the random forest algorithm used to predict noise was 82%. We managed to predict day 2 noise exposure for 250 participants, with the information from day 3 and day 4.

The non-wear time of all the sensor devices was verified with all the participants during the mobility survey over the phone and was deleted before the final processing steps to maintain the highest possible data integrity.

### 2.7. Other Covariates

Age in our analysis was coded as a continuous variable. Sex/gender (male vs. female) and couple and cohabitation status (in couple and cohabiting vs. not) were coded as binary variables. Monthly alcohol drinks (number of glasses of alcohol consumption monthly) and body mass index (BMI in kg/m^2^, from measured height and weight) were coded continuously. Annual household income was standardized by the number of units in the household, considering members younger than 14 years old as 0.5 units. After such an adjustment, three categories were created for household income based on tertiles: low, middle, and high. Other categorical variables were: the place of residence (Paris, close suburb, far suburb), education attainment (lower than Baccalaureat, equal to Baccalaureat, higher than Baccalaureat), and employment status (stable, unstable, unemployed, retired, and other). To control for the neighborhood socioeconomic effect on BP, winsorised living standards [34] in the residential area of the participants was adjusted for [13,23].

### 2.8. Statistical Analysis

#### 2.8.1. Linear Multilevel Models

We employed multi-pollutant multilevel models to assess the relationship of the air pollution exposure between the morning and evening measurements with the morning-to-evening BP change (with a random effect at the individual level). The models were weighted according to the time duration the between morning and evening BP measurements.

The potential confounders adjusted for in all the models were: age, sex, BMI, physical activity, alcohol consumption, weekday vs. weekend,, temperature, relative humidity, education [35], employment status, income, residence area [36], living standard of the residence area [37], noise, and percentage of time spent at home, out-of-home, and in motorized and non-motorized transport.

#### 2.8.2. Mixture Models

Moreover, we analyzed the joint effect of the morning-to-evening air pollutants exposure mixture (NO_2_, NO, CO, O_3_, and PM_2.5_) on the morning-to-evening BP change using quantile G-computation models adjusting for all the covariates mentioned above. It is one of the analytic approaches developed specifically for estimating the joint effect of exposure mixtures [22]. First, it transforms the exposures into quantized versions, i.e., cutting them into categorical variables in which the categories are created using quantiles of the exposures as cut points [22]. It then fits a linear model. This technique is an advanced version of the weighted quantile sum regression, relaxing the directional homogeneity, non-linearity, and non-additivity assumptions. The estimated coefficient *ψ* from the quantile G-computation model is interpretable as the change in the outcome attributed to increasing all the targeted exposures by one quantile (one quartile in our case) simultaneously. When there is directional heterogeneity across the associations between the mixture components and the outcome, positively associated components carry positive weights and negatively associated components negative weights. These weights are interpreted as the proportion of the positive or negative partial effect and are summing to 1.0, respectively [22].

#### 2.8.3. Sensitivity Analysis

**Figure 1:**
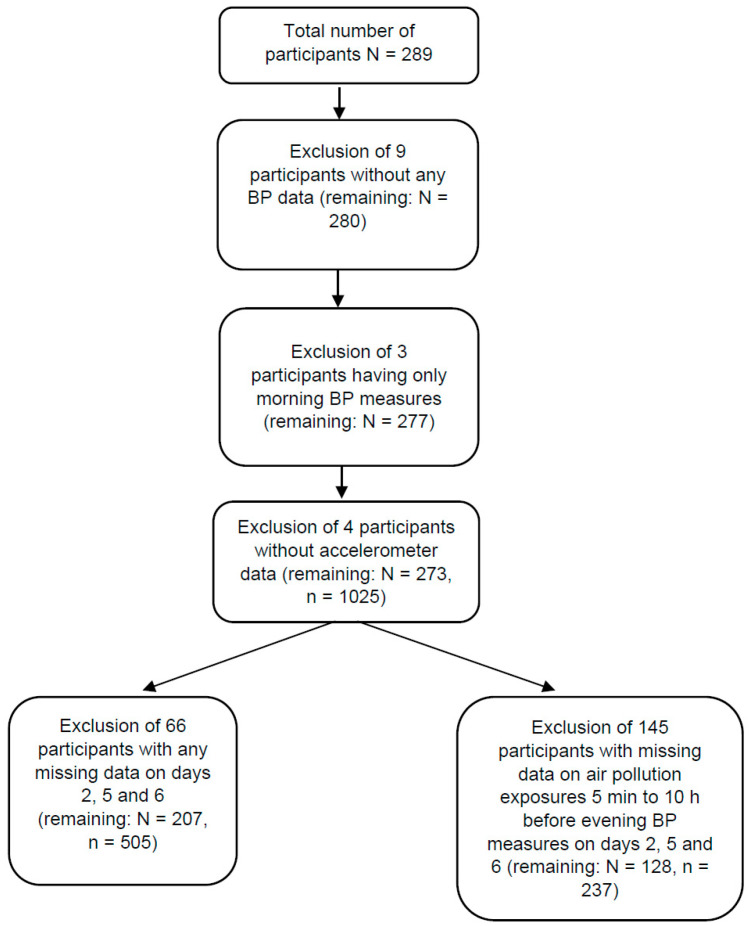
Data processing flow chart. n: number of morning-evening BP measurement pairs.

To capture the acute effect of pollutant exposures on changes in resting BP (morning to evening), we averaged air pollutant exposures over 5 min to 10 h before the evening BP measurements. While the main analysis was conducted in 207 participants, this particular sensitivity analysis was limited to 237 observations from 128 participants as we lost 145 individuals due to not having air pollution exposure data for those exposure windows of interest (Figure 1). We employed multi-pollutant multilevel models and quantile G-computation models to assess the associations between air pollution exposures at these different temporal scales and morning-to-evening BP changes.

## 3. Results

### 3.1. Population Characteristics

Among 207 participants, 57% were women, and the mean age was 50 (range: 33 to 67). The majority had an education higher than a Baccalaureat (71%), 24% stopped at the Baccalaureat, and the rest (5%) had a lower education. Sixty-eight percent had a stable job, 13% were retired, 2% were unemployed, and 4% had an unstable job. The participants mainly lived in the close suburbs of Paris (75%), only 24% lived in Paris, and 1% in the further suburbs. Seventeen individuals (8%) reported being diagnosed by a physician as hypertensive at some point in their lives. Thirteen of these seventeen participants were medicated for this hypertension. The mean morning SBP and DBP were 115.75 mmHg and 73.25 mmHg, while the mean evening SBP and DBP were 117.89 mmHg and 73.20 mmHg, respectively (Table 1). The change between morning and evening in SBP had a median of 2.0 (10th and 90th percentiles: −10.0, 14.0), while the corresponding values for DBP were 0.0 (−9.0, 8.8). Based on our previous publication [38], the hourly median NO_2_, O_3_, and PM_2.5_ concentrations reported by AirParif monitoring stations during our study time were 38.00 μg/m^3^, 46.90 μg/m^3^, and 11.90 μg/m^3^, which were relatively higher than the median concentrations recorded for this study (Table S2). We also examined the correlation between the accelerometry MVPA and the exposure to air pollutants over the period between the morning and the evening blood pressure measurements; none of the Pearson correlation coefficients were associated with a *p*-value < 0.05.

### 3.2. Linear Multilevel Models

The multi-pollutant models mainly showed small negative effects or no effect of the over-the-day exposure to NO_2_, NO, O_3_, and CO on the mean change in resting BP (SBP and DBP) from morning to evening, as shown in Table 2. For PM_2.5_ and NO_2_, the point estimates were positive, however, the 95% confidence interval included the null. On the contrary, CO exposure over the day was negatively associated with the morning-to-evening DBP changes (β = −0.09 mmHg; 95% CI: −0.18, −0.01) (Table 2).

### 3.3. Mixture Models

We did not observe any overall joint effect of the air pollutant mixture averaged over the day on morning-to-evening BP change. NO, O_3_, and PM_2.5_ were positive contributors in the mixture models, whereas, NO_2_ and CO were negative contributors to the overall association of the mixture (Table 3).

### 3.4. Sensitivity Analyses

**Figure 2:**
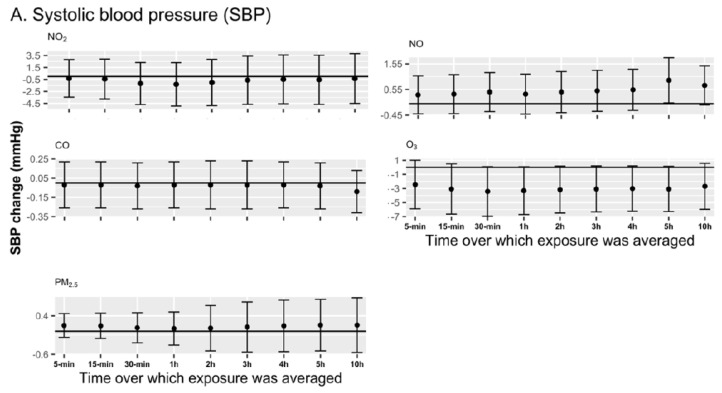

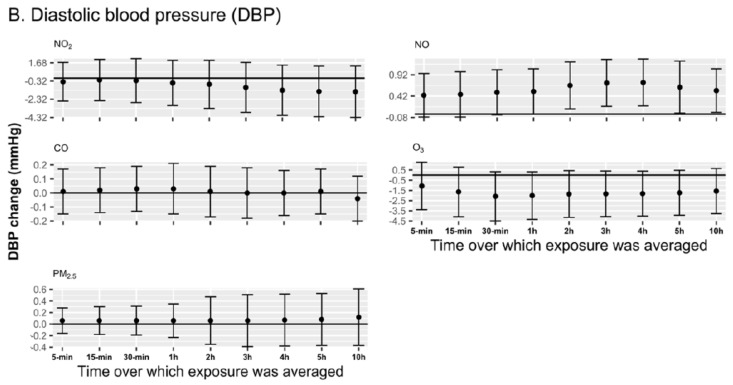
Associations between the personal exposure to air pollution concentrations and systolic blood pressure (SBP) (**A**) and diastolic blood pressure (DBP) (**B**). Figures represent the change in SBP or DBP from morning to evening (with 95% CIs) associated with a 10-ppb increase in the personal exposure to NO_2_, NO, and O_3_ or 100-ppb increase in CO, or 10-μg/m^3^ increase in PM_2.5_ averaged over 5 min to 10 h preceding the evening BP measurement; estimates from multi-pollutant multilevel models. MobiliSense Study, 128 participants, 237 pairs of morning and evening resting BP measurements.

NO exposure averaged over 2 to 10 h before the evening BP measure increased the morning-to-evening DBP by 0.63 mmHg (95% CI: 0.02, 1.24) to 0.74 mmHg (95% CI: 0.19, 1.29) (Figure 2B), while the magnitude of the association gradually decreased with the increase in the exposure period beyond 4 h. We found that pollutants such as NO_2_, CO, PM_2.5_, and O_3_ had no associations with BP (both SBP and DBP) across all exposure windows (from the previous 5 min to 10 h before measuring evening BP). In mixture models, among all exposure windows (see Table S3), the air pollutant mixture over the previous 5 min but also over the previous 2 h was positively associated with DBP, where a quartile increase in the air pollutants of the mixture for example during the 5 previous minutes increased the morning-to-evening DBP change by 4.38 mmHg (95% CI: 0.52, 8.24).

## 4. Discussion

We observed that the overall mixture of the five pollutants studied (NO, CO, NO_2_, O_3,_ and PM_2.5_) over the whole day did not affect resting SBP and DBP in this panel study of 207 healthy individuals living in the Grand Paris. When considering specific pollutants over the whole day, only CO had a weak negative association with the morning-to-evening BP change. However, in our sensitivity analysis, we found a positive relationship between the mixture of air pollutants over the previous 5 min but also 2 h and DBP. In the linear multilevel multi-pollutant models, we observed that NO exposure in the previous 2 h to 10 h before the evening measurement increased the morning-to-evening DBP change, where the strongest association was documented for the exposure averaged over the previous 4 h (β = 0.74 mmHg).

Previous studies have reported acute elevation in BP with higher daily levels of PM_2.5_ [15,39], or specifically increasing SBP (compared to a baseline) [40,41], while others found null associations [42,43]. One-hour to 24-h averaged exposures to concentrations of O_3_ increased BP [17,18,44] and the association was stronger during the cold weather period (October–December) [16]. However, we found no associations between O_3_ and BP, which may be due to the fact that our study population was relatively young (mean age of 51 years) and only 6% were hypertensive, while previous studies showed that people with pre-existing conditions are more susceptible to air pollution [45,46].

Regarding NO_2_, in previous studies, a short-term increase in NO_2_ with a 14-day lag (i.e., the exposure 14 days before the BP measurement) was associated with increased SBP but did not affect DBP [47], while another study documented that the 24-h averaged NO_2_ exposure was positively associated with SBP only in warm seasons (July–September) [16]. However, no association was observed with DBP in either warm or cold seasons (October–December) [16]. Moreover, some studies reported that short-term exposures to NO, CO, and O_3_ were associated with increased blood pressure [16,18,48], while others found the opposite [49] or no associations [45]. Nevertheless, due to the lack of studies that have applied mixture modeling methods in estimating the effect of air pollution on BP, and thus the inability to compare our findings with those obtained from other studies, our results must be interpreted with caution.

A potential reason that accounts for the inconsistencies between our findings and the findings from previous studies is the definition of the outcome; while our outcome was the change in the participants’ BP from morning to evening on the same day, the outcome considered in past studies was the mean difference in BP across participants. Another possible explanation for the discrepancies is the exposure time windows from minute level to several hours (before the evening BP measure) or a mean of 14 h for the exposure period between morning and evening measures considered in our study, which were shorter exposure windows than several short-term exposure studies considering day-lag exposure windows [36,42,43]. Another aspect to consider while comparing to the findings from past studies is the method of air pollution assessment. Most past studies assessed air pollution exposure indirectly, for instance, from the nearby monitoring stations to approximate personal exposure. Various studies have suggested that personal exposure to air pollutants may not induce the same responses in humans as ambient background levels due to differences in sources and chemical composition [50,51,52]. Moreover, the few studies that estimated multi-pollutant models often included only two pollutants [18,45,53,54], while we controlled for four other pollutants to estimate the effect of individual pollutants on BP. In summary, to this day, associations between exposure to individual air pollutants and BP are still difficult to interpret because of the variety of exposure levels and sources, the differences in the pollutants and confounders accounted for, the different assessment methods and exposure windows for air pollution, the reliance on clinical or ambulatory BP measurements, as well as differences between population susceptibility and regional contexts.

Also, from the same MobiliSense study, in a previous article, we used quantile G-computation models to investigate the relationship between exposure to air pollutants and ambulatory blood pressure (rather than resting blood pressure, measured with a different device, like in the present article) [13]. We found that exposure to a comparable mixture of air pollutants in the previous 5 min was related to a higher ambulatory SBP (exposures in longer time windows led to weaker associations), but not to a higher ambulatory DBP [13]. Even though the pollutants involved are the same, the finding from the present study that the air pollutants mixture over the previous five minutes increased DBP differs from the finding from the previous study. It may be because the previous study used ambulatory BP of participants measured every 30 min during the activities of the day [13], whereas the present study modeled change in resting BP from morning to evening.

Overall, our study did not document any associations, other than with CO in the main analysis and NO and the air pollution mixture on DBP in the sensitivity analyses. Given that previous studies have highlighted the significance of monitoring resting blood pressure for human health [14,21] and how air pollutants may affect resting blood pressure [15,18,19], future studies should aim at further understanding the temporal relationship of air pollution with resting BP, measuring exposures within time windows of various dimensions and applying repeated measurement of resting BP.

### 4.1. Strengths and Limitations

A primary strength of this study is that we could rely on repeated resting BP measurements over several days. Other strengths of this study include the measurement of personal exposures to air pollutants instead of using station-monitored measures. Using personal sensors is believed to be optimal for studying pollutant-induced health effects by limiting exposure measurement error [43] and potential exposure misclassification [55]. The sensors also measured personal meteorological parameters (temperature, relative humidity) and activity patterns (with the GPS receivers), which gave a better representation of each participant’s daily environment that could potentially influence their blood pressure and so confound the relationships of interest. Personal monitoring of a relatively large range of air pollutants and the use of a new statistical technique, quantile G-computation, permitted us to estimate the joint effect of an air pollutant mixture on changes in BP.

However, several limitations of the study should be noted. First, it is possible that the duration of air pollution exposures considered here may not be long enough to find measurable changes in resting BP since it was shorter than in several other short-term exposure studies of resting BP often considering day-level exposure windows [18,49,56]. Second, another limitation is that our sample size was relatively small and consequently the study lacked adequate statistical power. Therefore, some of the associations documented in our study should be interpreted cautiously, as they might be prone to type-I errors. Third, it is believed that circumstantial factors like anxiety, diet, circadian rhythm, mood, etc., could influence the BP measurements, especially when there are fewer repeated measurements recorded per participant. Fourth, importantly, our sample was biased toward older people with stable jobs, higher education, and living in the close suburbs of Paris, which could affect the generalizability of these results. Fifth, it would have been interesting to assess whether the air pollution effects differed among hypertensive participants, however, there were only 13 hypertensive participants in our sample, so we could not investigate this issue. Finally, it should be noted that our mixture modeling strategy jointly considering the associations of the exposure to different air pollutants over the day on the change in blood pressure assumed that all these air pollutants would influence blood pressure with the same time lag.

### 4.2. Conclusion

Overall, the combined mixture of the pollutants considered had a small positive or no effect on SBP and DBP change from morning to evening. When shorter time exposure windows were considered (from few minutes to few hours), both NO and the mixture showed positive associations with the morning-to-evening SBP change in some of the models. Future studies with a larger number of participants are required to test the associations between exposure to several air pollutants and resting BP at varying temporal scales of exposure from minutes to days.

## Figures and Tables

**Table 1:** Descriptive statistics for study participants.

Personal Characteristics	Mean (Standard Deviation) or N (%)	Median (2.5th Percentile, 97.5th Percentile)
Age	50.01 (8.84)	50 (35, 65)
Body mass index (kg/m^2^)	25.36 (6.38)	23.91 (18.64, 39.17)
Monthly alcohol consumption (number of times per month)	8.48 (8.99)	4.33 (0, 30.42)
Sex		
Woman	118 (57%)	NA
Men	89 (43%)	NA
Hypertension		
Yes	17 (8%)	NA
No	190 (92%)	NA
Education level		
Less than baccalauréat	11 (5%)	NA
Baccalauréat equivalent	50 (24%)	NA
Above baccalaureate	146 (71%)	NA
Employment		
Stable (permanent contract)	141 (68%)	NA
Unstable (temporary contract)	10 (4%)	NA
Retired	26 (13%)	NA
Unemployed	5 (2%)	NA
Other	25 (12%)	NA
Residence		
Paris	49 (24%)	NA
Close suburbs	156 (75%)	NA
Far suburbs	2 (1%)	NA
Marital status		
Unmarried	147 (71%)	NA
Married	69 (29%)	NA
Nationality		
French	201 (97%)	NA
Non French	6 (3%)	NA
Blood pressure measurements (mmHg)		
Morning SBP	115.75 (14.14)	114 (106, 126)
Evening SBP	117.89 (13.9)	117 (108, 128)
Morning DBP	73.25 (9.35)	72 (66, 79)
Evening DBP	73.2 (9.22)	74 (67, 79)

MobiliSense study: 207 participants, n = 503 observations. NA: Not applicable.

**Table 2:** Adjusted mean change (95% CI) in resting systolic blood pressure (SBP) and diastolic blood pressure (DBP) (mmHg) from morning to evening associated with a 10-ppb increase in personal exposure to air pollutants (NO_2_, NO, O_3_) or 100-ppb increase in CO or 10 μg/m^3^ increase in PM_2.5_ averaged over the duration between the morning and evening SBP measurements of the day; estimates from a multi-pollutants multilevel model.

Variables in the Model	Systolic Blood Pressure (SBP) Estimate (95% CI)	Diastolic Blood Pressure (DBP) Estimate (95% CI)
NO_2_	0.32 (−2.55, 3.19)	−1.13 (−3.12, 0.85)
NO	−0.35 (−0.81, 0.10)	−0.07 (−0.38, 0.24)
CO	−0.02 (−0.14, 0.11)	−0.09 (−0.18, −0.01)
O_3_	−0.84 (−2.88, 1.19)	−0.61 (−2.00, 0.78)
PM_2.5_	0.04 (−0.20, 0.29)	0.06 (−0.10, 0.23)
Age	−0.06 (−0.22, 0.10)	−0.05 (−0.16, 0.06)
Body mass index	−0.12 (−0.30, 0.05)	−0.08 (−0.20, 0.03)
Temperature	−0.24 (−0.68, 0.20)	−0.39 (−0.69, −0.09)
Relative humidity	−0.28 (−0.45, −0.11)	−0.09 (−0.21, 0.02)
Living standard of the residential area	0.12 (−0.01, 0.25)	0.06 (−0.03, 0.15)
Monthly alcohol drinks	−0.09 (−0.22, 0.04)	−0.07 (−0.16, 0.01)
Proportion of time out-of-home	−3.81 (−8.24, 0.61)	−1.01 (−4.10, 2.07)
Proportion of time in motorized transport	3.64 (−11.46, 18.73)	−7.77 (−18.30, 2.76)
Percentage of time physically active	−0.12 (−0.29, 0.04)	−0.10 (−0.22, 0.01)
Weekend (ref: weekdays)	−0.24 (−2.65, 2.16)	−0.17 (−1.86, 1.52)
Male (ref: female)	−0.83 (−3.24, 1.57)	−0.81 (−2.43, 0.81)
Residence (ref: Close suburbs)		NA
Far suburbs	0.05 (−10.67, 10.77)	−2.69 (−9.90, 4.52)
Paris	−0.02 (−2.70, 2.65)	−0.57 (−2.37, 1.23)
Employment status (ref: others)		NA
Employment (Retired)	1.70 (−2.89, 6.28)	0.34 (−1.50, 2.19)
Employment (Stable)	0.39 (−3.22, 4.00)	−1.81 (−5.46, 1.84)
Unemployed	4.36 (−3.25, 11.98)	NA
Employment (Unstable)	1.23 (−4.85, 7.32)	1.06 (−2.03, 4.14)
Education (ref: equivalent to baccalaureat)	NA	NA
Education higher than Baccalaureat	−0.21 (−2.95, 2.53)	1.99 (−3.14, 7.13)
Education lower than Baccalaureat	−2.22 (−7.63, 3.19)	−2.45 (−6.55, 1.66)
Tertile income (ref: high tertile income)	NA	NA
Medium tertile income	0.67 (−2.05, 3.39)	1.30 (−0.53, 3.13)
Low tertile income	2.17 (−0.53, 4.87)	1.28 (−0.54, 3.10)

MobiliSense Study, 207 participants, 505 pairs of morning and evening resting BP measurements. CI: confidence interval. All models were weighted for the time between the morning and evening measures and adjusted for age, sex, body mass index, physical activity, alcohol consumption, education, employment, household income per member, living standard of the residential area, temperature, relative humidity, noise, proportion of time spent out-of-home and in motorized transport, and week vs. weekend.

**Table 3:** Associations (95% CI) between a one quartile increase in exposure to a mixture of five air pollutants [averaged over the duration between morning to evening blood pressure measurements (BP)] and change in resting blood pressure from morning to evening; estimates from quantile G-computation.

	Systolic Blood Pressure		Diastolic Blood Pressure	
Air Pollutants	Coefficient β	Effect of Mixture *ψ* (95% CI)	Coefficient β	Effect of Mixture *ψ* (95% CI)
NO_2_	−0.79	−0.33 (−3.31, 2.65)	−0.27	−0.53 (−2.66, 1.60)
NO	0.30		0.43	
CO	−0.75		−1.46	
O_3_	0.12		0.23	
PM_2.5_	0.78		0.53	

MobiliSense Study, 207 participants, 505 pairs of morning and evening resting BP measurements. CI: confidence interval. All models were weighted for the time between the morning and evening measures and adjusted for age, sex, body mass index, physical activity, alcohol consumption, education, employment, household income per member, living standard of the residential area, temperature, relative humidity, noise, proportion of time spent out-of-home and in motorized transport, and week vs. weekend.

## Data Availability

Data will be made available on request.

## Supplementary Materials

The following supporting information can be downloaded at: https://www.mdpi.com/article/10.3390/ijerph22060872/s1, Section S1: Web appendix. Section S2: Descriptive statistics on pollutants. Section S3: Associations with air pollution mixture. References [27,57,58] are cited in the supplementary materials.
