# Supplementary material for: Exposure to Air Pollution and Changes in Resting Blood Pressure from Morning to Evening: The MobiliSense Study
Source: Int J Environ Res Public Health. 2025 May 31;22(6):872. doi: 10.3390/ijerph22060872 (PMC12193271; doi:10.3390/ijerph22060872)
Supplement: Supplementary file 1 [file ijerph-22-00872-s001.zip › ijerph-3429415-supplementary.pdf]

Section S1: Web appendix

Appendix 1: Calibration process of the PAM measurements

The PAM is an autonomous wearable platform that incorporates multiple sensors for physical and chemical parameters. The data are stored in a secure digital card inside the monitor and uploaded through a general packet radio service (GPRS) to a secure access FTP server.

A1.1 Gaseous pollutants

The PAM device contains small electrochemical (EC) sensors for the quantification of CO, NO, NO_2 and O_3 [1]. The sensor performance has been extensively characterised in laboratory conditions and in indoor and outdoor co-locations with reference instruments [1].

Out of 21 PAMs of the study, 3 PAMs were located on the roof of the Department of Chemistry (Cambridge University), outdoor next to the reference instruments. The three calibrated PAMs were then used as a "gold standard" to calibrate the rest of the 21 PAMs used in this study with an outdoor co-location in the local environment at the Medical Faculty of Sorbonne Université. A linear regression equation was applied to the outdoor co-location data to obtain the calibration parameters in order to convert the raw signals (mV) to mixing ratios (ppb) of the corresponding air pollutant concentration following the methodology described in Chatzidiakou et al. 2019 [2].

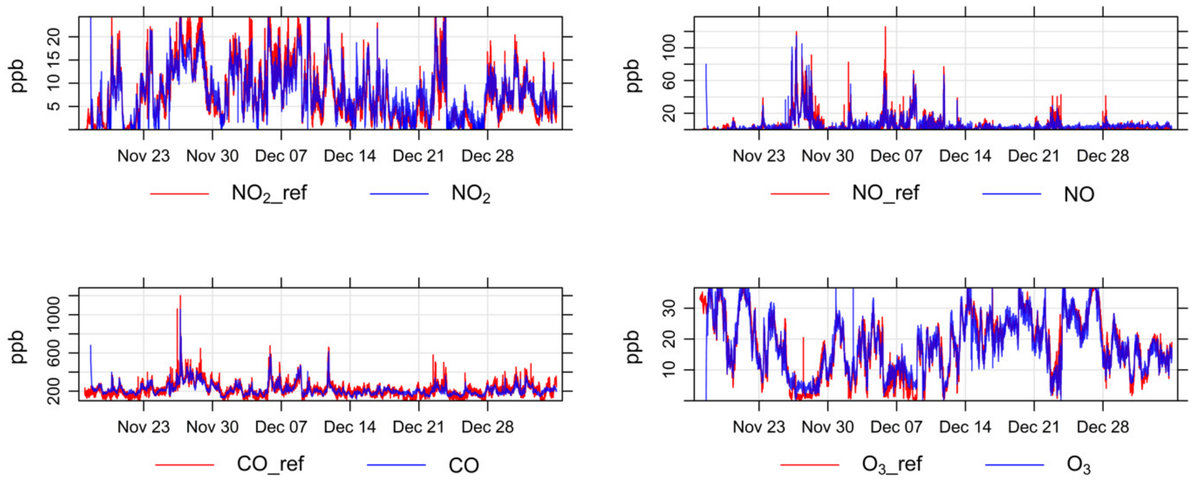

Figure S1. Outdoor co-location of one representative PAM with calibrated reference instruments at the Cambridge university at a 1 min time resolution.

Table S1. Performances of regression models used for the calibration of air pollution measurements conducted with PAM devices, following the methodology described in Chatzidiakou et al. 2019 [2]: the first 3 lines are based on outdoor co-location data collected in Cambridge while the 17 following lines are based on outdoor co-location data collected in Paris (see details on this two-step process explained in the footnote).

	Air pollutants							
	NO_2		NO		CO		O_3	
Device ID	RMSE	R^2	RMSE	R^2	RMSE	R^2	RMSE	R^2
222*	2.07	0.84	4.47	0.90	36.01	0.74	2.53	0.93
232*	1.52	0.62	1.74	0.94	35.31	0.78	2.22	0.94
245*	2.45	0.67	1.62	0.84	46.04	0.57	2.77	0.92
223	1.90	0.96	4.34	0.92	16.71	0.99	2.75	0.95
224	2.51	0.93	3.58	0.95	17.32	0.99	2.98	0.94
227	2.46	0.93	4.85	0.90	31.51	0.97	3.78	0.90
231	2.04	0.95	2.62	0.97	15.91	0.99	3.82	0.90
233	2.06	0.95	2.81	0.97	17.46	0.99	2.60	0.95
237	2.45	0.93	3.26	0.95	24.57	0.98	3.15	0.93
240	2.48	0.93	4.34	0.93	31.85	0.97	3.45	0.92
241	2.80	0.91	3.74	0.94	27.57	0.98	3.11	0.93
243	3.03	0.89	3.07	0.96	18.54	0.99	3.27	0.93
244	3.25	0.88	3.19	0.96	20.84	0.97	3.40	0.92
226	4.19	0.79	4.73	0.90	30.04	0.92	3.75	0.89
229	2.64	0.92	2.83	0.97	15.83	0.98	3.00	0.93
230	1.95	0.96	2.12	0.98	15.24	0.98	2.69	0.94
235	2.76	0.83	2.82	0.97	15.42	0.98	3.59	0.84
236	2.52	0.93	3.16	0.96	19.24	0.97	4.05	0.87
238	2.68	0.84	2.82	0.97	17.18	0.98	3.63	0.90
242	3.03	0.80	2.60	0.98	18.68	0.97	3.65	0.89

MobiliSense Study

ID: Identification number; RMSE: Root mean square error; R^2: Variability explained by the corresponding linear models

*The first 3 devices were co-located in Cambridge with a reference instrument. The reported models pertain to the relationships between this PAM measurements and the reference instrument measurement. The 17 following lines pertain to outdoor co-location data collected in Paris where the 3 previous devices were co-located with 17 other PAM devices. In these 17 regression models, the outcome was the predicted air pollution concentration from the 3 pre-calibrated PAMs using the calibration established based on the Cambridge data. The regression model related the measurement of each of the 17 PAMs with the predicted pollution level from the 3 pre-calibrated PAMs.

In the two steps, the model that was used was the same, to obtain the calibration parameters to convert the raw signals (mV) to mixing ratios (ppb) of the corresponding air pollutant concentration following the methodology described in Chatzidiakou et al. 2019 [2]. In brief, temperature effects were corrected through the auxiliary electrode, which might have a different sensitivity than the working electrode. The cross-sensitivities between the NO_2 and O_3 measurements were corrected via a specific parameter. As the CO and NO sensors were found to be sufficiently selective, the cross-sensitivity parameter was set to zero for the calibration of these gaseous pollutants.

A1.2 Particulate mass

Particulate matter was measured with a commercial miniaturized Optical particle counter (OPC) (Alphasense OPC-N2) integrated in the PAM that is capable of counting air particles of different diameters [2]. The OPC classifies air particles into 16 different bins according to the size of their diameter in the range of 0.38 to 17 μm.

Optical particulate matter (PM) sensors may overestimate PM mass due to the hygroscopic growth of particles when exposed to high relative humidity (RH) [3], which may result in differences in mass estimation compared to the reference gravimetric method.

We corrected for the effect of RH by applying an algorithm proposed by Di Antonio et al. [3], using a constant density of 1.65 g/cm^3 for the whole study period. A scaling factor for each sensor was estimated from a linear regression between RH-corrected $PM_{2.5}$ mass and the reference instrument measurements to correct for sensor-specific sensitivities.

References

1. Mead, M.I.; Popoola, O.A.M.; Stewart, G.B.; Landshoff, P.; Calleja, M.; Hayes, M.;Baldovi, J.J.; McLeod, M.W.; Hodgson, T.F.; Dicks, J.; et al. The use of electrochemical sensors for monitoring urban air quality in low-cost, high-density networks. *Atmos. Environ*. **2013**, *70*, 186–203.

2. Chatzidiakou, L.; Krause, A.; Popoola, O.A.M.; Di Antonio, A.; Kellaway, M.; Han, Y.; Squires, F.A.; Wang, T.; Zhang, H.; Wang, Q.; et al. Characterising low-cost sensors in highly portable platforms to quantify personal exposure in diverse environments. *Atmos. Meas. Tech.* **2019**, *12*, 4643–4657.

3. Di Antonio, A.; Popoola, O.; Ouyang, B.; Saffell, J.; Jones, R. Developing a Relative Humidity Correction for Low-Cost Sensors Measuring Ambient Particulate Matter. *Sensors* **2018,** *18*, 2790. http://www.mdpi.com/1424-8220/18/9/2790

Section S2: Descriptive statistics on pollutants.

Table S2. Descriptive statistics on pollutant exposures and BP measurements. Air pollution exposure averaged over several time windows prior to evening BP measurements.

	**Mean (Standard Deviation)**	**Median (2.5th percentile, 97.5th percentile)**
**NO_2 (PPB)**		
Five minutes	10.96 (4.63)	10.5 (2.18, 21.07)
Fifteen minutes	11.1 (4.36)	10.5 (3.65, 20.85)
Thirty minutes	11.21 (4.19)	10.7 (5, 20.3)
One hour	11.11 (4.1)	10.4 (5.09, 20.1)
Two hours	10.86 (3.84)	10.3 (4.58, 19.74)
Three hours	10.69 (3.73)	10.3 (4.66, 19.17)
Four hours	10.59 (3.69)	10 (4.55, 18.72)
Five hours	10.56 (3.72)	9.9 (4.49, 18.61)
Ten hours	10.43 (3.86)	9.8 (3.99, 19.25)
**NO (PPB)**		
Five minutes	20.69 (20.61)	13.2 (2.1, 87.1)
Fifteen minutes	20.06 (20.04)	12.5 (2.18, 84.12)
Thirty minutes	19.65 (19.94)	11.8 (2.1, 82.57)
One hour	19.18 (19.66)	11.2 (2.08, 78.13)
Two hours	18.26 (18.9)	10.7 (2, 74.67)
Three hours	17.79 (18.96)	10.7 (2.19, 71.9)
Four hours	17.55 (18.91)	10.8 (2.19, 67.99)
Five hours	17.52 (18.96)	10.6 (2.1, 70.8)
Ten hours	19.25 (21.4)	12 (2.97, 79.59)
**CO (PPB)**		
Five minutes	576.55 (594.48)	443.7 (180.43, 1711.99)
Fifteen minutes	572.49 (583.15)	454.5 (183.7, 1628.85)
Thirty minutes	568.71 (576.45)	445.8 (178.29, 1507.16)
One hour	564.69 (574.09)	444.8 (175.72, 1306.44)
Two hours	561.89 (574.09)	450.6 (175.1, 1328.99)
Three hours	564.21 (576.93)	450.6 (168.54, 1431.3)
Four hours	572.43 (583.6)	449 (158.39, 1448.2)
Five hours	583.32 (592.79)	467.4 (171.22, 1450.38)
Ten hours	656.24 (653.94)	490.3 (194.91, 2023.43)
**O_3 (PPB)**		
Five minutes	20.69 (20.61)	13.2 (2.1, 87.1)
Fifteen minutes	20.06 (20.04)	12.5 (2.18, 84.12)
Thirty minutes	19.65 (19.94)	11.8 (2.1, 82.57)
One hour	19.18 (19.66)	11.2 (2.08, 78.13)
Two hours	18.26 (18.9)	10.7 (2, 74.67)
Three hours	17.79 (18.96)	10.7 (2.19, 71.9)
Four hours	17.55 (18.91)	10.8 (2.19, 67.99)
Five hours	17.52 (18.96)	10.6 (2.1, 70.8)
Ten hours	19.25 (21.4)	12 (2.97, 79.59)
**$PM_{2.5}$ ($\mu g/m^3$)**		
Five minutes	13.96 (47.49)	3.8 (0, 88.4)
Fifteen minutes	13.37 (44.85)	3.9 (0, 94.31)
Thirty minutes	12.46 (39.42)	3.7 (0, 91.55)
One hour	11.35 (34.79)	3.6 (0, 96.31)
Two hours	10.13 (26.31)	3.5 (0, 93.07)
Three hours	9.72 (23.73)	3.4 (0, 87.81)
Four hours	9.62 (22.98)	3.3 (0, 82.8)
Five hours	9.62 (22.86)	3.3 (0, 80.11)
Ten hours	10.74 (22.41)	3.6 (0.47, 83.4)

Section S3: Associations with air pollution mixture.

Table S3. Associations (95% CI) between a one quartile increase in exposure to a mixture of five air pollutants averaged over 5 minutes to 10 hours prior to each evening blood pressure (BP) measurement and change in resting BP from morning to evening; estimates from G-computation*.

	Systolic BP		Diastolic BP	
**Air pollutants**	**Coefficient β**	**Effect of mixture ψ (95% CI)**	**Coefficient β**	**Effect of mixture ψ (95% CI)**
**Five minutes**				
NO_2	-0.17		-0.13	
NO	1.09		1.42	
CO	0.98	0.50 (-5.32, 6.31)	1.03	4.38 (0.52, 8.24)
O_3	-0.96		1.50	
$PM_{2.5}$	-0.45		0.56	
**Fifteen minutes**				
NO_2	-0.44		-0.25	
NO	0.46		0.09	
CO	1.26	1.20 (-5.15, 7.54)	1.56	3.32 (-0.95, 7.59)
O_3	0.09		1.15	
$PM_{2.5}$	-0.17		0.78	
**Thirty minutes**				
NO_2	-1.74		-0.72	
NO	1.96		0.87	
CO	0.4	-1.57 (-8.39, 5.26)	1.39	2.41 (-2.21, 7.03)
O_3	-1.01		0.93	
$PM_{2.5}$	-1.18		-0.05	
**One hour**				
NO_2	-2.4		-0.59	
NO	2.15		1.09	
CO	1.38	0.62 (-5.38, 6.63)	1.77	3.76 (-0.33, 7.85)
O_3	0.22		1.35	
$PM_{2.5}$	-0.73		0.14	
**Two hours**				
NO_2	-1.25		-0.58	
NO	1.66		0.51	
CO	1.85	4.30 (-3.12, 11.71)	2.37	5.15 (0.14, 10.16)
O_3	2.26		2.39	
$PM_{2.5}$	-0.22		0.46	
**Three hours**				
NO_2	-2.27		-1.78	
NO	0.68		-0.23	
CO	2.26	0.88 (-5.91, 7.67)	2.76	
O_3	0.21		1.12	2.64 (-1.90, 7.18)
$PM_{2.5}$	0.01		0.78	
**Four hours**				
NO_2	-1.59		-1.11	
NO	2.62		1.22	
CO	1.01	1.40 (-5.13, 7.94)	1.93	3.44 (-0.99, 7.87)
O_3	-0.23		1.40	
$PM_{2.5}$	-0.41		0.01	
**Five hours**				
NO_2	-0.56		-1.02	
NO	0.67		0.06	
CO	2.53	2.03 (-4.86, 8.91)	2.49	2.10 (-2.57, 6.78)
O_3	-0.06		0.60	
$PM_{2.5}$	-0.56		-0.03	
**Ten hours**				
NO_2	-0.97		-0.69	3.24 (-1.44, 7.93)

NO	1.07	0.32 (-6.55, 7.19)	-0.11
CO	1.97		2.56
O_3	-0.92		1.38
$PM_{2.5}$	-0.83		0.11

MobiliSense Study,128 participants, 237 pairs of morning and evening resting BP measurements
CI: confidence interval
* Exposure to all air pollutants averaged over the same corresponding time window were included in the same model.
All models were adjusted for age, sex, body mass index, physical activity, alcohol consumption, education, employment, household income per member, living standard of the residential area, temperature, relative humidity, noise, proportion of time spent out-of-home and in motorized transport, and week vs. weekend.
